# Diet Quality Associated with Total Sodium Intake among US Adults Aged ≥18 Years—National Health and Nutrition Examination Survey, 2009–2012

**DOI:** 10.3390/nu9111164

**Published:** 2017-10-25

**Authors:** Carla I. Mercado, Mary E. Cogswell, Cria G. Perrine, Cathleen Gillespie

**Affiliations:** 1 Division of Diabetes Translation, National Center for Chronic Disease Prevention and Health Promotion, Centers for Disease Control and Prevention, 4770 Buford Highway NE, Atlanta, GA 30341, USA; 2 Division for Heart Disease and Stroke Prevention, National Center for Chronic Disease Prevention and Health Promotion, Centers for Disease Control and Prevention, 4770 Buford Highway NE, Atlanta, GA 30341, USA; mec0@cdc.gov (M.E.C.); ckg2@cdc.gov (C.G.); 3 Division of Nutrition, Physical Activity, and Obesity, National Center for Chronic Disease Prevention and Health Promotion, Centers for Disease Control and Prevention, 4770 Buford Highway NE, Atlanta, GA 30341, USA; hgk3@cdc.gov

**Keywords:** sodium, diet, Healthy Eating Index 2010, and 24-h diet recall

## Abstract

Diet quality or macronutrient composition of total daily sodium intake (dNa) <2300 mg/day in the United States (US) is unknown. Using data from 2011–2014 NHANES (National Health and Nutrition Examination Survey), we examined 24-h dietary recalls (*n* = 10,142) from adults aged ≥18 years and investigated how diet composition and quality are associated with dNa. Diet quality was assessed using components of macronutrients and Healthy Eating Index 2010 (HEI-2010). Associations were tested using linear regression analysis adjusted for total energy (kcal), age, gender, and race/ethnicity. One-day dNa in the lower quartiles were more likely reported among women, older adults (≥65 years old), and lower quartiles of total energy (kcal) (*p*-values ≤ 0.001). With increasing dNa, there was an increase in the mean protein, fiber, and total fat densities, while total carbohydrates densities decreased. As dNa increased, meat protein, refined grains, dairy, and total vegetables, greens and beans densities increased; while total fruit and whole fruit densities decreased. Modified HEI-2010 total score (total score without sodium component) increased as dNa increased (adjusted coefficient: 0.11, 95% confidence interval = 0.07, 0.15). Although diet quality, based on modified HEI-2010 total score, increased on days with greater dNa, there is much room for improvement with mean diet quality of about half of the optimal level.

## 1. Introduction

Excess dietary sodium has been reported to be associated with adverse chronic conditions, such as hypertension, cardiovascular disease, and chronic kidney disease [1,2,3,4,5]. About 90% of US adults consume more sodium than the recommended US dietary guideline amount of less than 2300 mg/day [6,7]. US adults on average consume about 3500 mg/day of dietary sodium, excluding salt added at the table [8]. In the US, only about 6% of total sodium intake is estimated to come from salt added at the table [9]. The processed foods we eat contain the majority of sodium consumed, with 44% coming solely from 10 types of food and 65% from foods bought at a store [10]. Due to the ubiquitous presence of sodium in the US food supply, a modeling study suggested that it is difficult for adults who consume the recommended amount of sodium to meet other nutrient requirements [11]. Furthermore, since intra-individual variability in daily sodium intake can vary greatly (a difference of 897–1403 mg/day) [12], examining how macronutrient composition as well as diet quality are associated with sodium intake levels is needed. It is important to investigate if diet quality is better on days when less sodium is consumed. We know of no previous study that has examined diet quality or diet composition based on the sodium intake levels of that day. In other words, what is being eaten on days when meeting sodium intake recommendations versus exceeding them? 

Current US dietary guidelines provide advice on eating an overall balanced and healthy diet that promotes maintaining a healthy weight and preventing chronic diseases [7]. The Healthy Eating Index 2010 (HEI-2010) is a measure of diet quality and reflects the extent of adherence to the 2010 US Dietary Guidelines [13]. Overall, the HEI-2010 considers adequate consumption in reference to the total energy of fruits, vegetables, whole grains, dairy, protein, and fatty acids, while also considering consumption in moderation of refined grains, empty calories, and sodium. In 2003–2004, the mean estimated total HEI-2010 score in the US among those aged ≥2 years was 49.9 out of the maximum score of 100 [14]. 

With the amount of sodium in commonly consumed processed and restaurant foods, the possibility of adhering to US 2010 Dietary Guidelines and achieving sodium intakes of <2300 mg/day has been questioned. Using a United States (US) population representative sample of 24-h diet recalls, the objectives of this study were to: describe characteristics of US adults reporting one-day diet measures at each quartile of total daily sodium intake, investigate how a one-day diet composition based on macronutrients and HEI-2010 components varies at different levels of total daily sodium intake for that day, and compare HEI-2010 components and total scores for one-day diet measures with total daily sodium intake <2300 mg with those ≥2300 mg for that day. Since an individual’s sodium intake varies on a day-to-day basis [12], and diet quality associated with dietary sodium levels was examined, this study used a 24-h diet as the unit of interpretation, not an individual. We investigated diet quality based on sodium intake levels of that day given the abundant presence of sodium in the US food supply, not diet quality of individuals based on their usual daily sodium intake.

## 2. Materials and Methods

The National Health and Nutrition Examination Survey (NHANES) is designed to represent the health and nutrition status of the non-institutionalized US population at a given time using a complex, multi-stage, probability sampling design. Since 1999, NHANES has been a continuous survey with each cycle taking two years. Sampling design and data collection have been previously described [15]. Standardized interview administered 24-h diet recalls were collected using the Automated Multiple-Pass Method [16] initially in person during the examination visit with a second 24-hour diet recall collected by telephone 3 to 10 days later. Since intra-individual variation (or individual day-to-day variation) in sodium intake can be substantial and the objective is to investigate diet quality based on total daily sodium intake given the sodium content in the US food supply, this project focused on investigating the diet quality at the day level rather than the individual level. For this reason, we only used the initial in-person 24-h diet recall rather than both diet recalls to maintain independent observations.

Since dietary interpretation of this study was intended for US adults, 24-h diet recalls from participants 18 years old or older were included from 2011–2014 cycles of NHANES (*n* = 11,539) [17,18]. Sodium content for all foods and beverages, including water, for each 24-h diet recall was calculated by using the USDA’s Food and Nutrient Databases for Dietary Studies 2011–2012 and 2013–2014. Sodium from salt added at the table was not included in the calculation of total sodium. Response rates for corresponding NHANES cycles ranged from 69–70%. Diet recalls were excluded if incomplete or total energy was equal to zero (*n* = 1107), total daily sodium intake was greater or less than 2.5 standard deviations from the mean of the normal transformed distribution (*n* = 178), or if the participant was pregnant (*n* = 122). Numbers of those excluded are not mutually exclusive and final sample size was 10,142 24-h diet recalls.

The HEI-2010, a measure of diet quality based on following the 2010 Dietary Guidelines for Americans, comprises of 12 components, as shown in Table 1 [19]. Nine of the 12 components measure the consumption of adequate amounts of total fruit, whole fruit, total vegetables, greens and beans, whole grains, dairy, total protein foods, seafood and plant proteins, and fatty acids. The other three components take into account the moderate consumption of refined grains, sodium, and empty calories foods (Table 1). Empty calories include calories from discretionary solid fats and added sugars as well as from alcohol beyond moderate amount determined by 2010 US Dietary Guidelines of two drinks per day (threshold of 28 g of ethanol) [13,19]. Calculation of the HEI 2010 individual components and total score for each 24-h diet recall were performed using the HEI 2010 SAS (statistical analysis software) program from US Department of Agriculture, Center for Nutrition Policy and Promotion and methodology from the National Cancer Institute [20]. A modified total HEI 2010 score (range 0–0), which excluded the sodium component, was calculated by subtracting the sodium component score from the total HEI-2010 score and the modified score was used in the analysis testing the association with total daily sodium.

### 2.1. Statistical Analysis

Characteristics and demographics of those reporting 24-h diet recalls and by total daily sodium quartiles were described as percentages and assessed for differences across groups with Pearson’s chi-square test for: gender, age groups (18–44, 45–64, and ≥65 years), race/ethnicity (non-Hispanic white, non-Hispanic black, Mexican-American, other Hispanic, non-Hispanic Asian, and other race/multi-racial), family income to poverty ratio (<100%, 100–299%, 300–499%, and ≥500%), education for those aged ≥25 years (<high school diploma, high school diploma, some college, and college degree), body mass index (BMI) categories using BMI status variable (underweight (<18.5 kg/m^2^), normal (18.5–25 kg/m^2^), overweight (25–30 kg/m^2^), obese (≥30 kg/m^2^)), self-reported hypertension (yes/no), self-reported diabetes (yes/no), and total energy (kcal) quartiles. Mean diet densities for each macronutrient and fiber (g/1000 kcal) (protein, carbohydrates, fiber, and total fat), grain and protein food (oz/1000 kcal) (whole grains, refined grains, meat protein, and seafood/plant protein), vegetable/fruit and dairy food (cups/1000 kcal) (total vegetables, dark green vegetables/beans, total fruit, whole fruit, and dairy), empty calories (% calories from solid fats, alcohol, and added sugars of total kcal), and modified total HEI 2010 score for all 24-h diet recalls were estimated. We used multiple linear regression to test the association between each diet density, empty calories, and modified total HEI score 2010 with total sodium (mg) adjusted for total energy (kcal), age (years), gender, and race/ethnicity. We used stratified multiple linear regression to test the association between a modified total HEI score 2010 and total sodium (mg) and interactions across subgroups of age (18–44, 45–64, and ≥65 years), gender, race/ethnicity (non-Hispanic white, non-Hispanic black, Mexican-American, other Hispanic, non-Hispanic Asian, and other race/multi-racial), and total energy (kcal) quartiles. Means and 95% confidence intervals were estimated for each HEI 2010 component and total scores by 24-h diet recalls with total sodium <2300 mg and those ≥2300 mg. Multiple linear regression was used to test mean differences for HEI 2010 components and modified total scores between groups of total daily sodium <2300 mg and ≥2300 mg adjusted for total energy (kcal), age (years), gender, and race/ethnicity. Percentages of daily intakes with maximum scores for each HEI 2010 component and modified total scores were estimated for total daily sodium <2300 mg and ≥2300 mg. Statistical significance was denoted as a *p*-value less than 0.05. All of the analyses were performed using STATA 14.0 (StataCorp LP, College Station, TX, USA) and considered dietary sample weights and adjusted variance estimates to account for the complex sampling design. 

## 3. Results

Characteristics of those reporting 24-h diet recalls by quartiles of total daily sodium are presented in Table 2. One-day intakes of total sodium in the lower quartiles were more likely reported among women than men (*p*-value ≤ 0.001), older adults (≥65 years old) than younger ones (18–44 years old) (*p*-value ≤ 0.001), and those self-reporting having hypertension (*p*-value = 0.002) or diabetes (*p*-value = 0.002). Distribution in family income to poverty ratio (IPR; *p*-value = 0.03) and education (*p*-value = 0.001) significantly differed across quartiles of total daily sodium. When compared to the IPR and education distributions of all 24-h diet recalls, those with IPR < 300% and education level of high school diploma or less were more likely to report a one-day intake in the first quartile of total daily sodium (701–2276 mg/day). The majority of 24-h diet recalls reporting total energy in the first two quartiles were also categorized in the first two total sodium quartiles (*p*-value < 0.0001). 

Mean macronutrient densities (g/1000 kcal) were higher for protein, fiber, and total fat, and were lower for total carbohydrates at greater levels of total daily dietary sodium (mg) intake after adjusting for total energy intake, age, gender, and race/ethnicity (Table 3). Densities (oz/1000 kcal) of meat protein and refined grains were higher with greater levels of total daily sodium. There was no significant difference in whole grain or seafood and plant protein densities (oz/1000 kcal) by total daily sodium. Total vegetables, dark green vegetables/beans, and dairy densities (cups/1000 kcal) were higher with greater total daily sodium, whereas, fruit densities (cups/1000 kcal) were lower. After adjusting for total energy intake, age, gender, and race/ethnicity, mean modified total HEI-2010 score was higher with greater total daily sodium intake and the percent of total calories from empty calories was lower. For example, with a total daily energy intake of 2000 calories and an increase of 500 mg of sodium (equal to the sodium in almost 1/4 teaspoon of salt or about the amount in one 4 oz frozen cheese pizza slice), mean daily diet composition changed in protein (+4.8 g), carbohydrates (−5.0 g), fiber (+0.2 g), total fat (+1.3 g), meat protein (+0.5 oz), refined grains (+0.3 oz), total vegetables (+0.1 cups), greens and beans (+0.03 cups), total fruit (−0.07 cups), whole fruit (−0.04 cups), dairy (+0.04 cups), empty calories (−2.4% of total kcal), and modified total HEI-2010 score (+1.12 points). Although there were no significant interactions among gender and age subgroups, there was significant interaction in the association between the modified HEI-2010 total score with total daily sodium by race/ethnic subgroups and total energy quartiles (Table 4).

A comparison of mean components and modified total-HEI 2010 scores between groups of total daily sodium <2300 mg and ≥2300 mg were presented in Table 5. After accounting for total energy intake, age, gender, and race/ethnicity, 24-h diet recalls with total daily sodium <2300 mg had significantly greater mean scores for the components of total fruit and refined grains when compared to those with total daily sodium intake ≥2300 mg. Mean scores for total vegetables, greens and beans, dairy, total protein foods, and empty calories were greater among 24-h diet recalls with total daily sodium ≥2300 mg than those <2300 mg. Although absolute mean of modified total HEI-2010 score was greater for total daily sodium <2300 mg as compared to ≥2300 mg, the adjusted mean difference shows a greater mean modified total HEI-2010 score for total daily sodium ≥2300 mg than <2300 mg. Mean scores were not significantly different between total daily sodium <2300 mg and ≥2300 mg for the components of whole fruit, whole grains, seafood and plant protein, and fatty acid ratio. 

## 4. Discussion

Although days with sodium intake in lower quartiles when compared to those in higher quartiles were more likely reported by women, adults aged ≥65 years, those with IPR <300%, and those with education level of high school diploma or less; there was no difference in the amount of sodium intake across race/ethnic subgroups. Diet composition did change with increasing total sodium intake. While protein, fiber, and total fat densities were positively associated with total daily sodium intake, the increase of protein density associated with sodium intake were almost four times that of total fat and more than 20 times that of fiber. Therefore, it was not unexpected that meat density were positively associated with total daily sodium. Surprisingly, carbohydrate density were negatively associated with total daily sodium at similar magnitude to protein. This was probably driven by the negative association between fruit densities with total daily sodium intake. Other unexpected findings were the positive association between vegetable densities and the modified total HEI-2010 score with total daily sodium intake, while the percent of total calories coming from empty calories was negatively associated. 

Interestingly, the positive association between the modified total HEI-2010 score and total sodium intake differed within subgroups of race/ethnicity (interaction p-values comparing with non-Hispanic whites: 0.025 for non-Hispanic blacks and 0.005 for Mexican-Americans) and quartiles of total energy (interaction p-value comparing with 1st total energy quartile: 0.014 for 3rd quartile and <0.001 for 4th quartile). When compared to the association between the modified total HEI-2010 score and total sodium intake found in diet recalls from non-Hispanic whites, the magnitude of the association was greater for diet recalls among non-Hispanic blacks and smaller for those among Mexican-Americans. In Mexican-Americans, there was no significant association between the modified total HEI-2010 score and total sodium intake, meaning that total scores on average were similar for those with low sodium intake versus high sodium intake on those days. Although it is unclear as to why the race/ethnic differences were found across race/ethnic subgroups, there was a clear connection between the modified total score and total sodium intake based on daily total energy. The majority of diet recalls with total sodium in the 4th quartile were also ones with total energy in the 4th quartile. The positive association between modified total score and total sodium intake was greatest at the 1st quartile of total energy and continued to diminish with each proceeding quartile to a non-significant association at the 4th total energy quartile. Since there was no difference in the amount of total daily sodium intake reported, there were differences in reported total daily energy (*p*-value < 0.0001) across race/ethnic subgroups, which could be a part of the explanation for the observed interaction with the modified total HEI-2010 score.

Days with total sodium <2300 mg had overall greater percentages of max HEI-2010 component scores than days with ≥2300 mg. However, total daily sodium was highly driven by total energy intake, with mean total energy intake of 1329 kcals/day (95% confidence intervals (CI) = 1230–1359) for 24-h diet recalls representing <2300 mg of total sodium when compared to 2416 kcals/day (95% CI = 2387–2446) for those ≥2300 mg of total sodium. This indicates that on days when total daily sodium was <2300 mg it was likely due to substantially lower energy intake. When testing the linear association between diet quality and sodium intake, this study controlled for total energy intake by using density measures for diet categories (per 1000 kcals) as well as including the total calories of 24-h diet recalls in the regression model, making estimates unbiased to the quantity of calories consumed. This may be one reason why the direction of the association between sodium intake and modified total HEI-2010 score changed from the unadjusted to the adjusted model. Another explanation could be that most of the foods categorized under the HEI-2010 empty calories component are high in fat and sugar content, not necessarily in sodium, and the empty calories component (score range 0–20) has the potential to contribute the most to the total score than any other HEI-2010 components (score ranges: 0–5 or 0–10). When considering components like empty calories and the significant inverse association between the percent of total calories from empty calories and total sodium, it may be worthwhile to consider the absolute number of empty calories. Even though the percent difference from empty calories between 24-h diet recalls with total sodium ≥2300 mg and <2300 mg was not significant (0.8%, *p*-value = 0.07), the number of empty calories was significantly greater for total sodium ≥2300 mg than <2300 mg (difference = 305 kcals, *p*-value ≤ 0.001).

Overall, there was plenty of room for improvement in aspects of diet quality beyond total dietary sodium. The HEI-2010 total score maximum value of 100, which represents complete adherence to US 2010 Dietary Guidelines, was close to 50 for both groups of total sodium ≥2300 mg and <2300 mg. Since we found that the HEI-2010 total score was greater for 24-h diet recalls with total sodium ≥2300 mg than those <2300 mg, it remains unclear whether it is possible to improve mean HEI-2010 components and total scores at total sodium <2300 mg with today’s food supply. A recent study that evaluated the extent to which the US food supply aligns with the 2010 Dietary Guidelines by examining the trends in the overall food supply and for each HEI-2010 component found that the overall HEI-2010 score for the US food supply hovered at 50 from 1970 to 2010 [21], consistent with the 24-h diet recalls in this study. The US food supply HEI-2010 sodium score remained at zero, the lowest possible score, for most of the years from 1970 to 2010. Therefore, there is reasonable doubt that overall population improvement on HEI-2010 scores can be achieved, given the current food supply.

Interestingly, this study found that the 24-h diet recalls with total sodium in the first quartile (701–2276 mg) were more likely to be reported by US adults with IPR <300% or with education level less than or equivalent to a high school diploma. Although overall diet quality has typically been reported to be better among people with greater income and/or education attainment [22,23], sodium intake may not follow this same pattern. A current evaluation of diet cost and how it relates to HEI-2010 components and total scores found that most scores improved as diet cost (cost of food ($)/2000 kcal) increased [24]. There was no significant association between the components for dairy, total protein, and fatty acid ratio with diet cost. However, sodium was the only HEI-2010 component score inversely associated with diet cost, meaning that as diet cost increased sodium diet quality deteriorated even after accounting for total energy intake. 

There are limitations in this study. First, 24-h diet recalls are subject to recall or misreporting bias. However, we do not anticipate differential reporting of sodium intake based on diet quality and therefore we do not expect our estimates to be biased; Second, this study was designed to evaluate the average diet quality based on the total sodium of a one-day diet measure given the sodium content in the US food supply. Consequently, our inference in the association between sodium intake and diet quality given the sodium content in the US food supply are within the scope of that day, and are not based on the individual’s usual diet quality or their usual sodium intake even if individual’s dietary habits are influenced by a health condition (i.e., hypertension or diabetes). Subsequently, dietary patterns for diet recalls with sodium intakes <2300 mg in the present analyses might not reflect that of the usual dietary pattern of individuals with usual sodium intake levels <2300 mg. Additionally, it can be argued that sodium intake may be better captured by 24-h urine collections or all of the dietary measures by controlled trials where precise recipes are followed and amount of food eaten can be determined by weighing meals. In this study, the amount of sodium along with macronutrients and HEI-2010 food components were dependent on not only self-report 24-h diet recall but the databases from the Center for Nutrition Policy and Promotion are used to calculate amounts and scores. However, since the objective of this study was to estimate the diet quality associated with the sodium intake of that day based on average U.S. food supply and not person level estimates, the databases used in this study are appropriate because they comprise of average food and nutrient composition in the US food supply. Finally, we were unable to thoroughly evaluate diet quality based on total sodium ≤1500 mg due to the small percentage of the population with total sodium consumption ≤1500 mg. Nonetheless, we tested the association between diet quality and sodium intake as a continuous variable in the regression analyses (Table 3 and Table 4) and determined a significant association with some diet quality measures and sodium intake.

## 5. Conclusions

To the best of our knowledge, this study is the first to evaluate the association between diet quality and total sodium intake on a given day. It is also the first to test whether diet quality differs between total sodium <2300 mg than ≥2300 mg on a given day. Overall, the HEI-2010 scores were greater for more components as well as the composite score among the days with total sodium ≥2300 mg than those <2300 mg. Based on the findings of this study, diets on days consisting of total sodium ≥2300 mg can meet better overall diet quality than on days with total sodium <2300 mg. However, overall diet quality, including sodium intake, still needs to be vastly improved for all US adults. Potential contributions to improving overall diet quality among US adults include, but are not limited to, increasing the consumption of fresh fruits and vegetables that are naturally low in sodium, selecting healthier food options based on nutritional labels, and customizing food orders to healthier options when eating outside the home. However, reducing sodium content in the food supply from food manufacturers would improve population total daily sodium intake more rapidly than changes made at the individual level.

## Figures and Tables

**Table 1:** The 2010 Healthy Eating Index (HEI-2010) components and scoring standards.

HEI-2010 Component	Score	Standard for Maximum Score	Standard for Minimum Score
	*Higher score indicates greater consumption*		
Total fruit	0–5	≥0.8 cup equivalent/1000 kcal	No fruit
Whole fruit	0–5	≥0.4 cup equivalent/1000 kcal	No whole fruit
Total vegetables	0–5	≥1.1 cup equivalent/1000 kcal	No vegetables
Greens and beans	0–5	≥0.2 cup equivalent/1000 kcal	No dark-green vegetables, beans, or peas
Whole grains	0–10	≥1.5 ounces equivalent/1000 kcal	No whole grains
Dairy	0–10	≥1.3 cup equivalent/1000 kcal	No dairy
Total protein foods	0–5	≥2.5 ounces equivalent/1000 kcal	No protein foods
Seafood and plant proteins	0–5	≥0.8 ounces equivalent/1000 kcal	No seafood or plant proteins
Fatty Acids ^a^	0–10	(PUFAs + MUFAs)/SFAs ≥ 2.5	(PUFAs + MUFAs)/SFAs ≤ 1.2
	*Higher score indicates lower consumption*		
Refined grains	0–10	≤1.8 ounces equivalent/1000 kcal	≥4.3 ounces equivalent/1000 kcal
Sodium	0–10	≤1.1 g/1000 kcal	≥2.0 grams/1000 kcal
Empty calories ^b^	0–20	≤19% of energy	≥50% of energy
*Total HEI*-2010 *score*	0–100	*Sum of all components*	
*Modified total HEI*-2010 *score ^c^*	0–90	*Total HEI*-2010 *score without the sodium component*	

Table reproduced with minor edits from USDA’s HEI-2010 publication [17,18]. ^a^ Ratio of poly- and monounsaturated fatty acids (PUFAs and MUFAs) to saturated fatty acids (SFAs); ^b^ Empty calories include calories from discretionary solid fats and added sugars as well as from alcohol beyond moderate amount determined by 2010 US Dietary Guidelines of two drinks per day (threshold >28 g ethanol); ^c^ Modified total HEI-2010 score was calculated by subtracting the sodium component score from the Total HEI-2010 score.

**Table 2:** Characteristics of one-day dietary measures by dietary sodium quartiles for US adults aged ≥18 years—National Health and Nutrition Examination Survey, 2011–2014.

Characteristics	All 24-h Diet Recalls	Total Daily Sodium ^a^			
		Quartile 1	Quartile 2	Quartile 3	Quartile 4
		684–2295 mg	2296–3189 mg	3190–4366 mg	4367–10,134 mg
	*n* = 10,142	*n* = 2539	*n* = 2532	*n* = 2538	*n* = 2533
	% (95% CI)	% (95% CI)	% (95% CI)	% (95% CI)	% (95% CI)
Gender *					
Men	49.3 (48.0, 50.6)	30.2 (28.0, 32.6)	40.0 (36.9, 43.2)	51.0 (47.6, 54.3)	73.6 (71.4, 75.7)
Women	50.7 (49.4, 52.0)	69.8 (67.4, 72.0)	60.0 (56.8, 63.1)	49.0 (45.7, 52.4)	26.4 (24.3, 28.6)
Age group (years) *					
18–44	46.5 (43.9, 49.1)	38.7 (35.3, 42.3)	42.3 (38.5, 46.1)	47.5 (43.8, 51.1)	56.5 (52.4, 60.5)
45–64	35.8 (33.9, 37.7)	37.1 (33.9, 40.4)	37.1 (34.2, 40.1)	35.5 (32.2, 39.1)	33.6 (30.0, 37.4)
65+	17.7 (16.5, 19.0)	24.2 (21.6, 26.9)	20.6 (18.0, 23.5)	17.0 (15.1, 19.1)	9.8 (8.4, 11.6)
Race/Ethnicity					
Non-Hispanic White	65.8 (60.4, 70.7)	63.9 (57.3, 70.1)	67.1 (61.3, 72.4)	66.9 (61.3, 72.1)	65.0 (59.8, 69.8)
Non-Hispanic Black	11.5 (8.8, 14.9)	13.0 (10.0, 16.9)	10.4 (7.8, 13.6)	11.4 (8.4, 15.2)	11.5 (8.6, 15.0)
Mexican American	8.9 (6.6, 11.8)	8.6 (6.3, 11.5)	7.9 (5.4, 11.3)	8.3 (6.3, 10.9)	10.7 (7.7, 14.7)
Other Hispanics	5.9 (4.4, 7.9)	7.0 (4.8, 10.0)	6.2 (4.6, 8.4)	5.5 (4.0, 7.6)	5.1 (3.7, 6.9)
Non-Hispanic Asian	5.2 (4.2, 6.4)	5.2 (4.0, 6.7)	5.2 (4.3, 6.4)	5.0 (3.8, 6.6)	5.2 (4.0, 6.8)
Other Race/Multi-Racial	2.7 (2.1, 3.6)	2.3 (1.7, 3.2)	3.2 (2.2, 4.6)	2.9 (1.9, 4.4)	2.6 (1.7, 3.9)
Family income to poverty ratio (IPR) *					
<100%	16.2 (13.7, 19.0)	19.8 (16.5, 23.6)	13.7 (11.6, 16.2)	14.2 (11.7, 17.2)	17.3 (13.1, 22.5)
100–299%	33.6 (31.0, 36.2)	34.4 (30.3, 38.7)	34.4 (30.9, 38.1)	33.0 (29.7, 36.5)	32.5 (29.1, 36.2)
300–499%	21.1 (18.9, 23.4)	19.0 (16.6, 21.7)	21.3 (17.7, 25.4)	21.0 (18.6, 23.6)	22.9 (19.2, 27.0)
≥500%	29.2 (26.1, 32.4)	26.8 (23.1, 30.9)	30.6 (26.6, 34.8)	31.8 (28.0, 35.8)	27.3 (23.0, 32.2)
Education (among those aged 25 years or older) *					
<High school diploma	15.8 (13.5, 18.3)	19.6 (16.3, 23.5)	16.2 (13.4, 19.5)	12.6 (10.7, 14.7)	14.8 (12.3, 17.8)
High school diploma	21.0 (18.9, 23.2)	22.8 (19.8, 26.2)	19.9 (17.4, 22.8)	19.6 (16.9, 22.6)	21.7 (18.0, 25.9)
Some college	30.8 (29.0, 32.6)	30.6 (27.6, 33.7)	31.7 (28.5, 35.2)	30.3 (27.1, 33.8)	30.4 (27.5, 33.4)
≥College degree	32.5 (28.8, 36.4)	27.0 (22.8, 31.6)	32.1 (28.2, 36.3)	37.5 (33.2, 42.0)	33.1 (28.3, 38.2)
BMI categories **^b^**					
Underweight	1.7 (1.3, 2.1)	1.9 (1.3, 2.9)	1.3 (0.8, 2.1)	1.7 (1.2, 2.4)	1.8 (1.3, 2.7)
Normal	30.1 (28.3, 32.0)	32.0 (29.2, 35.0)	31.2 (28.1, 34.5)	30.4 (27.1, 33.9)	27.1 (24.0, 30.4)
Overweight	32.5 (30.7, 34.4)	30.9 (27.5, 34.5)	31.1 (28.6, 33.7)	33.3 (30.5, 36.2)	34.6 (31.4, 37.9)
Obese	35.7 (33.9, 37.5)	35.2 (32.2, 38.2)	36.4 (33.3, 39.6)	34.6 (31.4, 37.9)	36.4 (33.1, 39.9)
Self-reported hypertension *	32.7 (30.7, 34.6)	36.4 (34.0, 38.9)	32.9 (30.0, 35.7)	31.8 (28.6, 35.0)	29.9 (27.5, 32.4)
Self-reported diabetes *	9.4 (8.7, 10.1)	11.4 (10.0, 12.8)	10.7 (8.8, 12.6)	7.8 (6.3, 9.3)	7.9 (6.6, 9.2)
Total energy (kcal) quartiles *^,c^					
1st quartile	22.2 (21.2, 23.2)	64.8 (62.2, 67.2)	22.0 (19.8, 24.5)	5.4 (4.3, 6.7)	1.1 (0.6, 1.9)
2nd quartile	25.3 (23.9, 26.7)	26.3 (24.3, 28.4)	43.3 (40.6, 46.1)	27.2 (24.6, 29.9)	5.2 (4.2, 6.3)
3rd quartile	26.6 (25.2, 28.0)	7.8 (6.1, 10.0)	28.0 (25.5, 30.6)	42.8 (39.4, 46.3)	25.9 (23.8, 28.1)
4th quartile	26.0 (24.9, 27.0)	1.1 (0.6, 2.1)	6.7 (5.4, 8.3)	24.7 (22.9, 26.6)	67.8 (65.8, 69.8)

* Pearson’s Chi-square *p*-value <0.05 testing difference across sodium quartiles; ^a^ Total daily sodium excludes sodium from salt added at the table; ^b^ BMI (body mass index) categories: underweight (<18.5 kg/m^2^), normal (18.5–25 kg/m^2^), overweight (25–30 kg/m^2^), obese (≥30 kg/m^2^); ^c^ Total energy quartiles (median (range)): 1st quartile (1155 kcal (186–1444)); 2nd quartile (1692 kcal (1445–1949)); 3rd quartile (2245 kcal (1950–2622)); and 4th quartile (3211 kcal (2623–8918)).

**Table 3:** Unadjusted and adjusted ^a^ linear regression coefficients from analyses of total sodium ^b^ (per 100 mg/day) with each macronutrients and HEI-2010 components (dependent variables)—National Health and Nutrition Examination Survey, 2011–2014 (*n* = 10,142).

Dependent Variables	Mean (95% CI)	Unadjusted Models		Adjusted Models ^a^	
		Coefficient ^c^ (95% CI)	*p*-Value	Coefficient ^c^ (95% CI)	*p*-Value
Macronutrients					
Protein (g/1000 kcal)	39.61 (39.02, 40.19)	0.077 (0.055, 0.100)	<0.001	0.481 (0.439, 0.524)	<0.001
Carbohydrates (g/1000 kcal)	121.22 (120.36, 122.08)	−0.352 (−0.399, −0.306)	<0.001	−0.504 (−0.575, −0.434)	<0.001
Fiber (g/1000 kcal)	8.46 (8.27, 8.66)	−0.033 (−0.042, −0.025)	<0.001	0.022 (0.007, 0.036)	0.004
Total fat (g/1000 kcal)	37.52 (37.21, 37.83)	0.117 (0.104, 0.129)	<0.001	0.131 (0.094, 0.168)	<0.001
HEI-2010 components					
Whole grains (oz/1000 kcal)	0.46 (0.44, 0.49)	−0.005 (−0.006, −0.004)	<0.001	−0.001 (−0.003, 0.001)	0.290
Meat protein (oz/1000 kcal)	3.00 (2.93, 3.07)	0.009 (0.007, 0.012)	<0.001	0.047 (0.042, 0.053)	<0.001
Seafood and plant protein (oz/1000 kcal)	0.77 (0.71, 0.82)	−0.002 (−0.004, 0.001)	0.238	−0.001 (−0.006, 0.003)	0.586
Refined grains (oz/1000 kcal)	2.61 (2.56, 2.66)	0.011 (0.008, 0.013)	<0.001	0.025 (0.022, 0.029)	<0.001
Total vegetables (cups/1000 kcal)	0.79 (0.76, 0.82)	−0.001 (−0.003, 0.0002)	0.096	0.014 (0.012, 0.017)	<0.001
Greens and beans (cups/1000 kcal)	0.11 (0.10, 0.12)	−0.0003 (−0.001, 0.0001)	0.149	0.003 (0.002, 0.003)	<0.001
Total fruit (cups/1000 kcal)	0.47 (0.44, 0.49)	−0.008 (−0.009, −0.007)	<0.001	−0.007 (−0.008, −0.005)	<0.001
Whole fruit (cups/1000 kcal)	0.34 (0.32, 0.36)	−0.007 (−0.008, −0.006)	<0.001	−0.004 (−0.005, −0.003)	<0.001
Dairy (cups/1000 kcal)	0.74 (0.72, 0.77)	0.002 (0.001, 0.003)	0.001	0.004 (0.002, 0.005)	<0.001
Empty calories (% of total kcal) ^d^	28.00 (27.47, 28.53)	0.005 (−0.015, 0.026)	0.597	−0.241 (−0.278, −0.205)	<0.001
Modified total HEI-2010 score ^e^	47.12 (46.36, 47.88)	−0.061 (−0.086, −0.036)	<0.001	0.112 (0.074, 0.151)	<0.001

Abbreviation: CI, confidence interval. ^a^ Regression models adjusted for total kilocalories, age, gender, and race/ethnicity (non-Hispanic white, non-Hispanic black, Mexican-American, other Hispanic, non-Hispanic Asian, and other race/multi-racial); ^b^ Total sodium excludes sodium from salt added at the table; ^c^ Linear regression coefficient for total sodium (independent variable). Each macronutrient and HEI-2010 component was analyzed in its own model as the dependent variable; ^d^ Empty calories include calories from discretionary solid fats and added sugars as well as from alcohol beyond moderate amount determined by the 2010 US Dietary Guidelines of 2 drinks per day (threshold > 28 g ethanol); ^e^ Modified total HEI-2010 score was calculated by subtracting the sodium component score from the Total HEI-2010 score (Table 1).

**Table 4:** Stratified unadjusted and adjusted **^a^** linear regression coefficients (β) from analyses of total sodium ^b^ (per 100 mg/day) with modified total HEI-2010 score ^c^ (dependent variables)—National Health and Nutrition Examination Survey, 2011–2014.

Stratification Characteristics	Sample Size	Modified Total HEI-2010 Score ^c^ Mean (95% CI)	Unadjusted Models		Adjusted Models ^a^		Interaction *p*-Value ^e^
			Coefficient ^d^ (95% CI)	*p*−Value	Coefficient ^d^ (95% CI)	*p*-Value	
All	1,0142	47.12 (46.36, 47.89)	−0.061 (−0.086, −0.036)	<0.001	0.112 (0.074, 0.151)	<0.001	n/a
Age group (years)							
18–44	4698	44.38 (43.59, 45.16)	−0.023 (−0.052, 0.006)	0.113	0.128 (0.079, 0.176)	<0.001	ref
45–64	3329	48.40 (47.38, 49.42)	−0.059 (−0.108, −0.011)	0.019	0.096 (0.022, 0.171)	0.013	0.051
65+	2115	51.75 (50.41, 53.08)	0.001 (−0.075, 0.078)	0.973	0.092 (−0.010, 0.194)	0.076	0.875
Gender							
Men	4989	46.13 (45.34, 46.93)	−0.052 (−0.078, −0.026)	<0.001	0.093 (0.048, 0.137)	<0.001	ref
Women	5153	48.08 (47.18, 48.98)	−0.036 (−0.083, 0.012)	0.138	0.150 (0.079, 0.222)	<0.001	0.497
Race/Ethnicity							
Non-Hispanic White	4118	47.39 (46.33, 48.46)	−0.066 (−0.099, −0.033)	<0.001	0.120 (0.069, 0.172)	<0.001	ref
Non-Hispanic Black	2364	44.25 (43.01, 45.49)	−0.016 (−0.056, 0.025)	0.439	0.186 (0.117, 0.256)	<0.001	0.025
Mexican American	1229	45.02 (43.76, 46.28)	−0.114 (−0.148, −0.079)	<0.001	0.003 (−0.073, 0.078)	0.941	0.005
Other Hispanics	957	47.49 (46.24, 48.74)	−0.048 (−0.093, −0.003)	0.039	0.071 (−0.040, 0.181)	0.201	0.545
Non-Hispanic Asian	1160	54.45 (53.12, 55.78)	0.009 (−0.051, 0.069)	0.762	0.087 (0.002, 0.177)	0.055	0.953
Other Race/Multi-Racial	314	44.88 (41.98, 47.79)	−0.106 (−0.217, 0.006)	0.062	0.197 (−0.061, 0.455)	0.130	0.352
Total energy (kcal) quartiles							
first	2495	48.91 (47.71, 50.11)	0.285 (0.167, 0.403)	<0.001	0.292 (0.170, 0.414)	<0.001	ref
second	2573	49.10 (47.93, 50.26)	0.189 (0.080, 0.298)	0.001	0.174 (0.077, 0.270)	0.001	0.257
third	2573	46.94 (46.13, 47.75)	0.071 (0.001, 0.140)	0.046	0.085 (0.026, 0.145)	0.007	0.014
fourth	2501	43.86 (42.80, 44.91)	−0.035 (−0.085, 0.014)	0.157	−0.031 (−0.084, 0.023)	0.254	<0.001

Abbreviation: CI, confidence interval. ^a^ Regression models adjusted for total kilocalories, age, gender, and race/ethnicity (Mexican-Americans, other Hispanics, non-Hispanic white, non-Hispanic black, and other race/multi-racial); ^b^ Total sodium excludes sodium from salt added at the table; ^c^ Modified total HEI-2010 score was calculated by subtracting the sodium component score from the Total HEI-2010 score (Table 1); ^d^ Linear regression coefficient for total sodium. Each macronutrient and HEI-2010 component was analyzed in its own model as the dependent variable; ^e^ Interaction between total sodium and each categorical descriptive characteristic were tested. *p*-value for the interaction term in the regression model are presented.

**Table 5:** Mean Healthy Eating Index-2010 component and modified total scores ^a^ and percent with maximum scores by total daily sodium intake ^b^ ≤2300 mg and >2300 mg for all 24-h diet recalls—National Health and Nutrition Examination Survey, 2011–2014 (*n* = 10,142).

	24-h Diet Recalls					
Component (Maximum Score)	Total Daily Sodium Intake ^b^ <2300 mg (*n* = 2551)		Total Daily Sodium Intake ^b^ ≥2300 mg (*n* = 7591)		Adjusted Score Difference ^c^ (95% CI)	*p*-Value for Difference
	Mean Score (95% CI)	% w/max Score	Mean Score (95% CI)	% w/max Score		
Total vegetables (5)	2.78 (2.69, 2.88)	25%	2.92 (2.85, 3.00)	22%	0.54 (0.41, 0.66)	<0.001
Greens and beans (5)	1.08 (0.97, 1.19)	17%	1.28 (1.21, 1.35)	18%	0.37 (0.24, 0.50)	<0.001
Total fruit (5)	2.37 (2.24, 2.51)	32%	1.92 (1.87, 2.06)	19%	−0.27 (−0.45, −0.08)	0.006
Whole fruit (5)	2.27 (2.10, 2.44)	38%	1.96 (1.87, 2.05)	27%	−0.11 (−0.31, 0.09)	0.256
Whole grains (10)	2.89 (2.67, 3.10)	12%	2.58 (2.42, 2.75)	8%	0.16 (−0.11, 0.43)	0.234
Dairy (10)	4.68 (4.52, 4.84)	17%	5.24 (5.10, 5.38)	16%	0.49 (0.30, 0.67)	<0.001
Total protein foods (5)	3.83 (3.75, 3.90)	49%	4.25 (4.20, 4.30)	59%	0.60 (0.50, 0.69)	<0.001
Seafood and plant protein (5)	1.97 (1.83, 2.11)	29%	2.07 (1.96, 2.18)	29%	−0.002 (−0.20, 0.20)	0.982
Fatty acid ratio (10)	5.45 (5.22, 5.68)	30%	5.14 (5.02, 5.25)	21%	−0.08 (−0.38, 0.23)	0.613
Sodium (10)	6.32 (6.14, 6.50)	26%	3.70 (3.60, 3.80)	5%	−4.48 (−4.66, −4.30)	<0.001
Refined grains (10)	6.90 (6.70, 7.10)	42%	6.11 (5.97, 6.25)	28%	−1.05 (−1.31, −0.79)	<0.001
Empty calories (20) ^d^	13.03 (12.53, 13.52)	26%	13.61 (13.35, 13.87)	21%	2.45 (1.92, 2.98)	<0.001
Modified total HEI-2010 score (90) ^a^	47.24 (46.08, 48.41)	0.1%	47.08 (46.34, 47.82)	0%	3.08 (1.86, 4.31)	<0.001

^a^ Modified total HEI-2010 score was calculated by subtracting the sodium component score from the Total HEI-2010 score (Table 1); ^b^ Total daily sodium intake excludes sodium from salt added at the table; ^c^ Difference (component score for total daily sodium intake ≥2300 mg minus those for total daily sodium intake <2300 mg) estimated by multiple linear regression adjusted for total caloric intake, age, gender, and race/ethnicity; ^d^ Empty calories include calories from discretionary solid fats and added sugars as well as from alcohol beyond moderate amount determined by the 2010 US Dietary Guidelines of 2 drinks per day (threshold > 28 g ethanol).
